# Development of a cognitive function marker based on D-amino acid proportions using new chiral tandem LC-MS/MS systems

**DOI:** 10.1038/s41598-020-57878-y

**Published:** 2020-01-21

**Authors:** Ren Kimura, Hisashi Tsujimura, Masaru Tsuchiya, Satoko Soga, Noriyasu Ota, Atsushi Tanaka, Hunkyung Kim

**Affiliations:** 1 Analytical Science Laboratories, Kao Corporation, 2606 Akabane, Ichikai-machi, Haga-gun, Tochigi 321-3497 Japan; 2 Biological Science Laboratories, Kao Corporation, 2606 Akabane, Ichikai-machi, Haga-gun, Tochigi 321-3497 Japan; 3 Analytical Science Laboratories, Kao Corporation, 1334 Minato, Wakayama-shi, Wakayama 640-8580 Japan; 4 Research Team for Promoting Independence of the Elderly, Tokyo Metropolitan Institute of Gerontology, 35-2 Sakae-cho, Itabashi-ku, Tokyo 173-0015 Japan

**Keywords:** Biomarkers, Diseases, Health care, Signs and symptoms

## Abstract

The incidence of dementia, a clinical symptom characterized by severe cognitive decline, is increasing worldwide. Predictive biomarkers are therefore required for early identification and management. D-amino acids in the brain contribute to cognitive function and are suggested as useful biomarkers for diagnosing dementia risk. To clarify their relationship with human cognitive decline, we developed an identification method of chiral metabolomics for detecting slight differences in chiral amino acid amounts. Chiral tandem liquid chromatography-tandem mass spectrometry systems were applied for sensitive and selective amino acid species along with chiral species determination based on anion and zwitterion exchange mechanisms. In a comprehensive health cohort (cross-sectional study), we measured blood chiral amino acid levels from 305 women (65–80 years old) classified into Control, Mild-cognitive-Impairment (MCI), and Dementia groups using the Mini-Mental State Examination. MCI exhibited higher D-Pro (D-Pro/(D-Pro + L-Pro)) proportion vs the Control group, suggesting this proportion as a useful biomarker for MCI. Biomarker accuracy was improved in combination with D-Ser proportion. Receiver operating characteristics analysis of the Control vs. MCI proportion obtained area under the curve (0.80) with 70% sensitivity and 84% specificity at the optimal cutoff value (0.30). Thus, dementia monitoring can be improved by including trace D-amino acids measurements.

## Introduction

Dementia is a clinical symptom indicating a type of cognitive dysfunction that, when severe, leads to increasing dependence on others in aspects of daily living. Various types of dementia include Alzheimer (AD), cerebrovascular, Lewy body, and frontotemporal dementia. The detailed cause of onset has not yet been elucidated and no effective biomarkers are available for the early diagnosis of dementia or selection of therapeutic agents^1^. The prodromal stage of dementia and mild cognitive impairment (MCI) have attracted attention in recent years as early stages of cognitive decline. It has been reported that approximately 10% of MCI converts to AD annually^2^. Moreover, 14–44% of patients with MCI improve to the status of healthy elderly individuals through early interventions^3^. Therefore, the early diagnosis of dementia risk and timely interventions, such as nutrition, exercise, and sleep, are desired to prevent cognitive decline in the at-risk elderly population.

Current methods of diagnosis require a wide range of clinical tests including cognitive performance assessment, genetic screening, measurement of amyloid beta and tau proteins in the liquor cerebrosinalis, brain imaging, and behavioural assessments^4^. Especially in recent years, numerous researchers have aimed to identify appropriate markers measurable in the blood for the early identification and management of dementia. For example, quantitative measurements of amyloid beta and its sequester protein in the blood have been developed^5–9^. However, these biomarkers are difficult to apply toward practical dementia monitoring owing to the high cost and low throughput nature of the analysis.

D-amino acids, which constitute relatively minor components compared to their L-enantiomers in biological processes, are present in microorganisms, plants, mammals, and humans and play specific biological functions. In the brain of mammals, D-serine (D-Ser), known as an agonist of N-methyl-D-aspartate receptors, is suggested to be associated with the saccharometabolism of astrocytes in early cognitive decline^10^. The increased expression of serine racemase is associated with aging and neuroinflammation, which likely contributes to the neurocognitive deficit observed in neurodegenerative diseases like AD^11^. Therefore, we assumed that the enantiomeric proportion of D-amino acids have the potential to be a novel candidate biomarker for dementia monitoring. Moreover, several functions of D-amino acids have been recently clarified with the advance of analytical technologies^12–16^. Therefore, a method for the identification of chiral amino acid metabolomics and the simultaneous separation of both molecular species and enantiomers is expected to be applicable to the study of the detailed functions of amino acids. In general, chiral compounds are currently analysed using liquid chromatography (LC) because of its robustness, flexibility, and wide availability. For example, 4-fluoro-7-nitro-2,1,3-benzoxadiazole (NBD-F) derivatives of chiral amino acid are quantified using a two-dimensional LC method with a reversed-phase column (first dimension: molecular species separation) combined with a chiral column (second dimension: enantiomer separation)^17,18^. Furthermore, a one-dimensional LC method using a chiral column without derivatisation for chiral amino acid analysis has also been reported^19^. Nevertheless, the two-dimensional method is time-consuming as a diagnostic assay as it requires complicated processes owing to the necessity of switching valves and incorporation of multiple loops. In comparison, although the one-dimensional method is applicable as a diagnosis assay, simultaneous separation of both molecular species and enantiomers (e.g., complete enantiomer separation of secondary amines such as racemic proline (DL-Pro)) has not been achieved and the low-precision of chiral amino acid analysis has been noted^19^. In addition, it is often difficult to simultaneously separate all proteinogenic chiral amino acids (especially, DL-Pro) by capillary electrophoresis-mass spectrometry^20^.

In this study, in order to evaluate the ability to provide early indication of cognitive disease risk through the use of promising biomarkers, a new method to quantify chiral amino acids was developed. This analytical technique should enable us to analyse chiral amino acids simultaneously within 30 min per sample, which is at least 10 times shorter than two-dimensional LC method^18^, indicating that the method has sufficient selectivity for clinical use. In order to obtain a new biomarker for early diagnosis of cognitive decline using chiral amino acids, correlation analysis between the amount of chiral amino acids in human blood and cognitive function was conducted in a comprehensive health cohort.

## Results

### Analytical method development for chiral amino acids using chiral tandem liquid chromatography-tandem mass spectrometry (LC-MS/MS)

#### Optimization of MS parameters and separation conditions

As only trace amounts of D-amino acids are present in biological samples and the detection of each D-amino acid is inhibited by various compounds such as peptides and proteins^17^, a highly sensitive and highly selective detection technology is required for simultaneous analysis of chiral amino acids. We first attempted to develop an analytical method using MS/MS, which enabled the detection of trace amounts of D-amino acids. In doing so, several MS parameters for detecting trace D-amino acids were optimized. Six-Aminoquinolyl-*N*-hydroxysuccinimidyl carbamate (AQC) derivatisation, which contains an amino pyridyl group with high ionization efficiency, was selected to detect D-amino acids at sub-femtomole levels. We found that a protonated molecule (precursor ion) and AQC-derived ion (product ion) were useful in multiple reaction monitoring (MRM) mode. In addition, as the strength of product ion was strongly influenced by collision energy (CE), optimized MS parameters including CE could be summarized (Table 1).

**Table 1:** Optimized MRM transitions of AQC-derivatised chiral amino acids.

Molecular Species	Q1	Q3	DP	EP	CE	CXP	Time
**Lys**	487	171	71	10	41	10	100
**Orn**	473	171	81	10	41	10	100
**Trp**	375	171	61	10	33	20	100
**Tyr**	352	171	116	10	31	10	100
**Cit**	346	171	11	10	37	18	100
**Arg**	345	171	46	10	41	26	100
**Phe**	336	171	11	10	29	14	100
**His**	326	171	36	10	25	22	100
**Met**	320	171	96	10	29	18	100
**Glu**	318	171	81	10	29	10	100
**Gln**	317	171	56	10	35	10	100
**Asp**	304	171	76	10	29	22	100
**Asn**	303	171	81	10	25	14	100
**Ile**	302	171	86	10	27	16	100
***allo*-Ile**	302	171	86	10	27	16	100
**Leu**	302	171	86	10	27	16	100
**Cys**	292	171	71	10	27	10	100
**Thr**	290	171	61	10	27	20	100
***allo*-Thr**	290	171	61	10	27	20	100
**Val**	288	171	91	10	27	18	100
**Pro**	286	171	61	10	25	16	100
**Ser**	276	171	61	10	27	18	100
**d4-Ala**	264	171	1	10	27	18	100
**Ala**	260	171	1	10	27	18	100
**Gly**	246	171	51	10	27	18	100

Multiple Reaction Monitoring: MRM; Q1 Mass: Precursor ion (*m/z*); Q3 Mass: Product ion (*m/z*); De-clustering Potential: DP (V); Entrance Potential: EP (V); Collision Energy: CE (V); Collision Cell Exit Potential: CXP (V); Time (msec).

**Figure 1:**
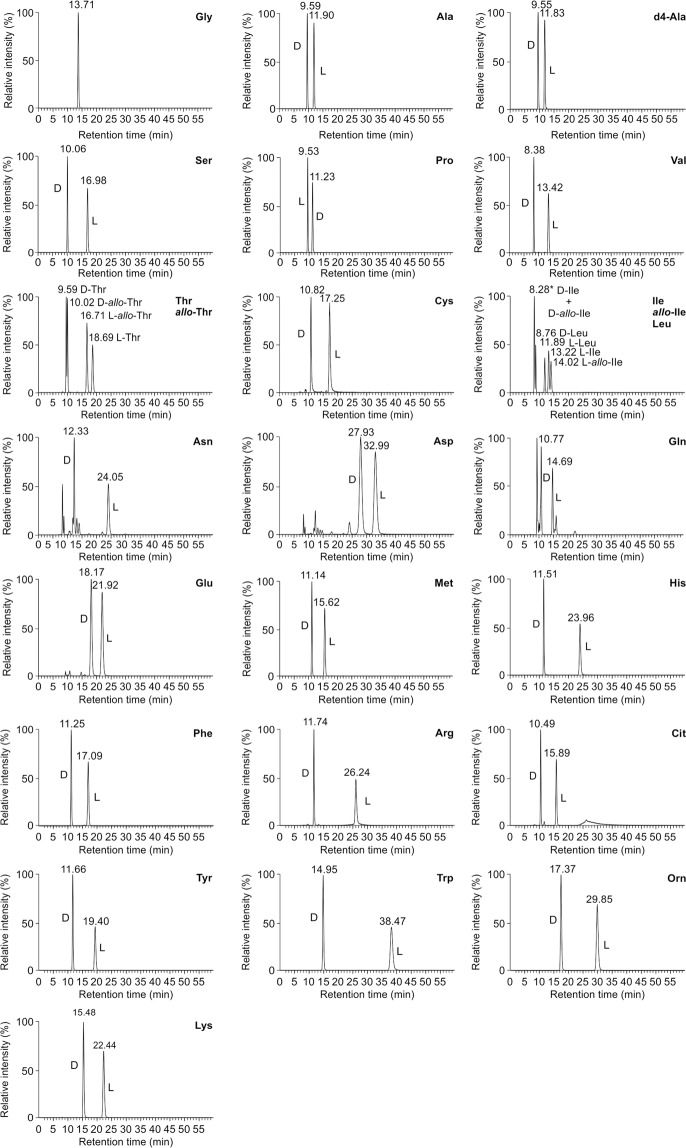
Chromatograms of chiral amino acid standards obtained using chiral tandem LC-MS/MS. *The separated spectra for D-Ile and D-*allo*-Ile are shown in Supplementary Fig. 1.

However, amino acid species are present along with their chiral species. To achieve the simultaneous separation of both molecular species and enantiomers, we attempted to develop chromatographic techniques using the MS/MS conditions. Focusing on the optimization of separation conditions, we achieved such separation by constructing chiral tandem LC systems using two kinds of ion-exchange type chiral columns (cinchona alkaloid-based chiral stationary phases) in a methanol solvent. A weak anion-exchange-type chiral column and a zwitterion-exchange-type chiral column were connected in series for detecting separate chiral amino acids. A weak anion-exchange-type chiral column (QN-AX column) was selected to obtain successful separation of molecular species. The molecular species of amino acids were eluted in the order of basicity, neutrality, and acidity, whereas they were not retained in reversed-phase conditions using methanol solvent. In turn, complete enantiomer separation was achieved through use of a zwitterion-exchange type chiral column (ZWIX (+) column), whereas good separation of molecular species was not achieved through the chiral stationary phase of zwitteic-exchangers, unlike the results from anion-exchangers. The simultaneous analysis of chiral amino acids: DL-alanine (Ala), DL-d4-Ala, DL-arginine (Arg), DL-asparagine (Asn), DL-aspartic acid (Asp), DL-cysteine (Cys), DL-glutamine (Gln), DL-glutamic acid (Glu), DL-histidine (His), DL-isoleucine (Ile), DL-leucine (Leu), DL-lysine (Lys), DL-methionine (Met), DL-phenylalanine (Phe), DL-proline (Pro), DL-serine (Ser), DL-threonine (Thr), DL-tryptophan (Trp), DL-tyrosine (Tyr), DL-valine (Val), DL-citrulline (Cit), DL-*allo*-Ile, DL-ornithine (Orn), DL-*allo*-Thr, and glycine (Gly), except for the separation between D-Ile and D-*allo*-Ile, was thus achieved via chiral tandem LC-MS/MS (Fig. 1). D-amino acids of primary amines eluted earlier than their enantiomers, whereas L-amino acids of a secondary amine, such as proline, eluted earlier than its enantiomers. In contrast, it was difficult to differentiate between D-Ile and D-*allo*-Ile using the developed methods. Therefore, to separate these molecular species, a chiral column-in-series (QD-AX and ZWIX (−)) method was required (Supplementary Fig. 1). The present chiral tandem LC-MS/MS system was a promising tool for the simultaneous analysis of chiral amino acids (L-amino acids and trace D-amino acids) within short time, although some peaks could be only partially separated.

#### Evaluation of chiral tandem LC-MS/MS

We evaluated the developed chiral tandem LC-MS/MS systems for range and linearity of the calibration curve, limit of detection, and relative standard deviation (Table 2). The range and linearity of the calibration curve were assessed using standard mixtures at concentrations of 0.0001, 0.001, 0.01, 0.1, 1, and 5 nmol/mL. Linearity was evaluated according to the correlation coefficient (r^2^). The maximum range of 0.0001–5 nmol/mL was shown for 33 compounds (DL-Trp, DL-Tyr, DL-Cit, DL-Met, DL-Glu, DL-Ile, DL-*allo*-Ile, DL-Leu, DL-Cys, DL-Thr, DL-*allo*-Thr, DL-Val, DL-Pro, DL-Ser, DL-d4-Ala, DL-Ala, and Gly) and the minimum range of 0.01–5 nmol/mL was shown for three compounds (DL-Arg, D-Gln). Linearity of all compounds was r^2^ > 0.99 (total average: 0.9994), indicating that the system maintained good linearity of the data. The limit of detection, expressed as signal-to-noise ratio of 3:1, was mainly 0.0001–0.01 nmol/mL, indicating that the system was sufficiently sensitive to detect trace amounts of D-amino acids. The relative standard deviation was obtained from the peak areas of 0.1 nmol/mL standards, which were evaluated in three successive analyses. Our results showed that the system maintained high repeatability, indicating that the values were within 0.1 and 9.4% (total average: 3.5%) and less than 20%.

**Table 2:** Performance evaluation of chiral tandem LC-MS/MS.

Molecular Species	Enantiomer	Chiral column	RT	Range	r^2^ value	RSD	LOD
			(min)	(nmol/mL)		(%)	(nmol/mL)
**Lys**	D	QN-AX + ZWIX(+)	15.48	0.001–5	1.0000	4.9	0.001
	L	QN-AX + ZWIX(+)	22.44	0.001–5	1.0000	7.1	0.001
**Orn**	D	QN-AX + ZWIX(+)	17.37	0.001–5	0.9999	4.3	0.001
	L	QN-AX + ZWIX(+)	29.85	0.001–5	1.0000	3.4	0.001
**Trp**	D	QN-AX + ZWIX(+)	14.95	0.0001–5	0.9999	5.1	0.0001
	L	QN-AX + ZWIX(+)	38.47	0.0001–5	1.0000	2.2	0.0001
**Tyr**	D	QN-AX + ZWIX(+)	11.66	0.0001–5	0.9999	1.2	0.0001
	L	QN-AX + ZWIX(+)	19.40	0.0001–5	1.0000	5.4	0.0001
**Cit**	D	QN-AX + ZWIX(+)	10.49	0.0001–5	0.9999	9.4	0.0001
	L	QN-AX + ZWIX(+)	15.89	0.0001–5	1.0000	3.2	0.0001
**Arg**	D	QN-AX + ZWIX(+)	11.74	0.01–5	0.9999	3.8	0.01
	L	QN-AX + ZWIX(+)	26.24	0.01–5	1.0000	1.5	0.01
**Phe**	D	QN-AX + ZWIX(+)	11.25	0.001–5	0.9995	1.7	0.001
	L	QN-AX + ZWIX(+)	17.09	0.001–5	0.9997	1.5	0.001
**His**	D	QN-AX + ZWIX(+)	11.51	0.001–5	1.0000	6.4	0.001
	L	QN-AX + ZWIX(+)	23.96	0.001–5	1.0000	2.4	0.001
**Met**	D	QN-AX + ZWIX(+)	11.14	0.0001–5	0.9993	1.6	0.0001
	L	QN-AX + ZWIX(+)	15.62	0.0001–5	0.9997	2.3	0.0001
**Glu**	D	QN-AX + ZWIX(+)	18.17	0.0001–5	0.9999	0.9	0.0001
	L	QN-AX + ZWIX(+)	21.92	0.0001–5	1.0000	4.0	0.0001
**Gln**	D	QN-AX + ZWIX(+)	10.77	0.01–5	1.0000	9.0	0.01
	L	QN-AX + ZWIX(+)	14.69	0.001–5	0.9998	8.1	0.001
**Asp**	D	QN-AX + ZWIX(+)	27.93	0.001–5	0.9999	5.7	0.001
	L	QN-AX + ZWIX(+)	32.99	0.001–5	0.9999	7.2	0.001
**Asn**	D	QN-AX + ZWIX(+)	12.33	0.001–5	0.9994	2.3	0.001
	L	QN-AX + ZWIX(+)	24.05	0.001–5	1.0000	1.6	0.001
**Ile**	D	QD-AX + ZWIX(−)	12.31	0.0001–5	0.9990	3.2	0.0001
	L	QN-AX + ZWIX(+)	13.22	0.0001–5	0.9989	2.2	0.0001
	D-*allo*-	QD-AX + ZWIX(−)	12.73	0.0001–5	0.9980	4.6	0.0001
	L-*allo*-	QN-AX + ZWIX(+)	14.02	0.0001–5	0.9991	1.6	0.0001
**Leu**	D	QN-AX + ZWIX(+)	8.76	0.0001–5	0.9995	7.8	0.0001
	L	QN-AX + ZWIX(+)	11.89	0.0001–5	0.9992	2.3	0.0001
**Cys**	D	QN-AX + ZWIX(+)	10.82	0.0001–5	0.9989	1.7	0.0001
	L	QN-AX + ZWIX(+)	17.25	0.0001–5	0.9982	1.3	0.0001
**Thr**	D	QN-AX + ZWIX(+)	9.59	0.0001–5	0.9990	2.7	0.0001
	L	QN-AX + ZWIX(+)	18.69	0.0001–5	0.9998	1.0	0.0001
	D-*allo*-	QN-AX + ZWIX(+)	10.02	0.0001–5	0.9990	9.4	0.0001
	L-*allo*-	QN-AX + ZWIX(+)	16.71	0.0001–5	0.9993	0.9	0.0001
**Val**	D	QN-AX + ZWIX(+)	8.38	0.0001–5	0.9960	0.6	0.0001
	L	QN-AX + ZWIX(+)	13.42	0.0001–5	0.9993	1.5	0.0001
**Pro**	D	QN-AX + ZWIX(+)	11.23	0.0001–5	0.9989	2.9	0.0001
	L	QN-AX + ZWIX(+)	9.53	0.0001–5	0.9979	2.3	0.0001
**Ser**	D	QN-AX + ZWIX(+)	10.06	0.0001–5	0.9993	5.0	0.0001
	L	QN-AX + ZWIX(+)	16.98	0.0001–5	0.9997	0.8	0.0001
**d4-Ala**	D	QN-AX + ZWIX(+)	9.55	0.0001–5	0.9999	3.3	0.0001
	L	QN-AX + ZWIX(+)	11.83	0.0001–5	0.9999	4.5	0.0001
**Ala**	D	QN-AX + ZWIX(+)	9.59	0.0001–5	0.9980	0.1	0.0001
	L	QN-AX + ZWIX(+)	11.90	0.0001–5	0.9977	1.6	0.0001
**Gly**	—	QN-AX + ZWIX(+)	13.71	0.0001–5	0.9983	4.3	0.0001

RT: Retention time; RSD: Relative standard deviation; LOD: Limit of detection.

### Cognitive function and correlational analysis of blood chiral amino acid proportions in community-dwelling elderly individuals

#### Cognitive function assessments

Based on the Mini-Mental State Examination (MMSE) score, 305 subjects were classified into Control (*N* = 150), MCI (*N* = 149), and Dementia (*N* = 6) groups, with an average score of 28.8 ± 0.1, 25.0 ± 0.1, and 16.3 ± 1.6, respectively. The average age in the respective groups was 74.5 ± 0.4, 73.9 ± 0.4, and 74.5 ± 2.0 years. No significant differences were noted in average age among the three groups. In addition, we investigated which cognitive category declined earlier by analysing the individual MMSE scores in the MCI group (Supplementary Fig. 2). We found that attention, recall, and understanding categories were associated with early cognitive decline.

#### Blood component analysis

**Figure 2:**
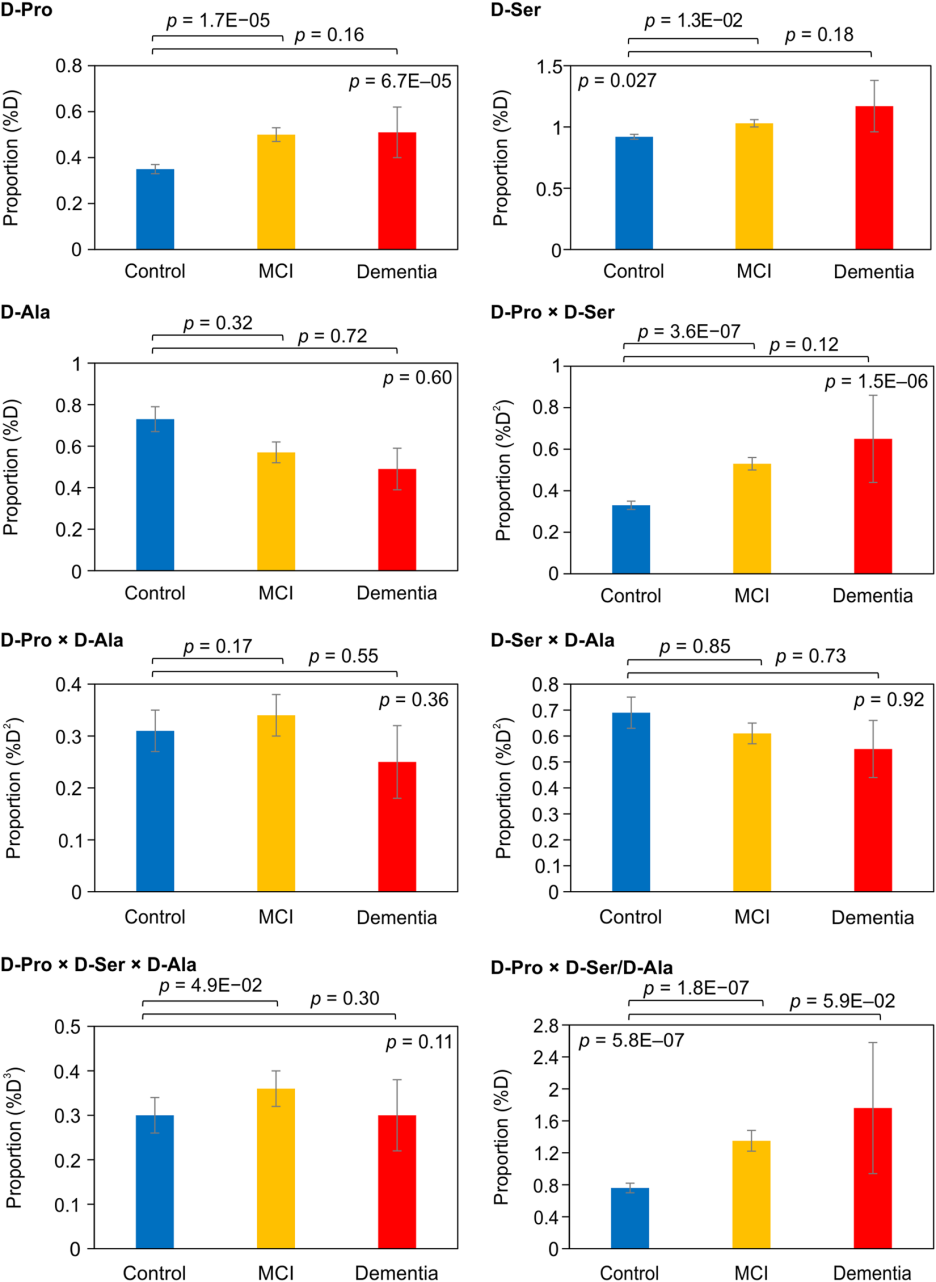
Blood enantiomeric proportions obtained in the Control, MCI, and Dementia groups.

In all 305 women (65–80 years old: Control, MCI, and Dementia groups), the blood components obtained by complete blood count could be determined (Table 3). No significant difference was observed in the main blood components among the three groups using the Kruskal-Wallis test. Moreover, blood chiral amino acid levels were analysed and three free D-amino acids (D-Pro, D-Ser, and D-Ala) were only detected on the chromatograms without interference peaks. The concentration of D-Pro (corresponding L-Pro) in the Control, MCI, and Dementia group was 0.46 ± 0.02 (140.0 ± 12.4) μM, 0.65 ± 0.03 (130.0 ± 13.0) μM, and 0.50 ± 0.10 (102.3 ± 13.2) μM, respectively. The concentration of D-Ser (corresponding L-Ser) in the Control, MCI, and Dementia group was 1.15 ± 0.02 (126.0 ± 2.0) μM, 1.26 ± 0.02 (120.4 ± 2.1) μM, and 1.36 ± 0.20 (119.4 ± 8.9) μM, respectively. The concentration of D-Ala (corresponding L-Ala) in the Control, MCI, and Dementia group was 1.80 ± 0.02 (250.2 ± 12.4) μM, 1.45 ± 0.12 (250.0 ± 5.0) μM, and 1.37 ± 0.36 (280.0 ± 34.4) μM, respectively. Figure 2 shows the blood enantiomeric proportion [D/(D + L) × 100] (%D) in each group. The proportion of D-Pro in the Control, MCI, and Dementia group was 0.35 ± 0.02, 0.50 ± 0.03, and 0.51 ± 0.11, respectively. The D-Pro proportion in the MCI group was significantly higher than that in the Control group. The proportion of D-Ser in the Control and MCI group was 0.92 ± 0.02 and 1.03 ± 0.03, respectively, with the latter being significantly higher (*p* = 1.3E−02). In contrast, no significant difference was observed in the proportion of D-Ala between the groups. Moreover, the proportion of D-Ser combined with that of D-Pro (multiplied proportion: D-Pro × D-Ser), the multiplied proportion combining D-Pro, D-Ser, and D-Ala (D-Pro × D-Ser × D-Ala), and the multiplied proportion (D-Pro × D-Ser) divided by the proportion of D-Ala (D-Pro × D-Ser/D-Ala) in the MCI group were significantly higher than those in the Control group, respectively. Notably, the D-Pro × D-Ser multiplied proportion significantly correlated with the MMSE score (Supplementary Fig. 3). Pearson’s coefficient showed a significant relationship between the two parameters (r = −0.258, *p* = 5.0E−06). The blood enantiomeric proportion was more associated with cognitive decline than L-amino acids levels. No significant difference was observed by only L-amino acids, which are abundant in biological samples.

**Table 3:** Blood components obtained in the Control, MCI, and Dementia groups.

Biomarker	Control	MCI	Dementia	*p-*value
	(N = 150)	(N = 149)	(N = 6)	
**CRE, mg/dL**	0.7 ± 0.01	0.7 ± 0.02	0.7 ± 0.06	0.4283
**T-CHO, mg/dL**	225.9 ± 2.9	224.2 ± 2.6	233.2 ± 26.4	0.9613
**HDL-CHO, mg/dL**	68.9 ± 1.5	68.7 ± 1.4	57.8 ± 4.8	0.2265
**TG, mg/dL**	151.9 ± 6.8	152.7 ± 6.7	173.7 ± 51.0	0.9139
**ALB, g/dL**	4.3 ± 0.02	4.3 ± 0.02	4.3 ± 0.1	0.4293
**WBC, /μL**	6022.0 ± 118.2	5892.6 ± 104.3	6250.0 ± 631.3	0.8430
**RBC, ×10**^**4**^**/μL**	431.9 ± 3.0	429.8 ± 3.1	414.3 ± 23.6	0.3489
**Hb, g/dL**	13.2 ± 0.1	13.1 ± 0.1	12.9 ± 0.5	0.5428
**Ht, %**	41.7 ± 0.3	41.7 ± 0.3	41.0 ± 1.7	0.8520
**Plt, ×10**^**4**^**/μL**	24.2 ± 0.4	24.5 ± 0.5	21.4 ± 1.7	0.2684
**MCV, fl**	96.7 ± 0.4	97.1 ± 0.4	99.7 ± 3.0	0.5911
**MCH, pg**	30.6 ± 0.2	30.6 ± 0.1	31.2 ± 0.7	0.6488
**MCHC, %**	31.7 ± 0.07	31.6 ± 0.06	31.3 ± 0.4	0.2293
**HbA1c, %**	5.5 ± 0.04	5.6 ± 0.06	6.0 ± 0.4	0.7679

CRE: creatinine; MCI: mild-cognitive-impairment; T-CHO: total cholesterol; TG: triglyceride; ALB: albumin; WBC: white blood cell; RBC: red blood cell; Hb: haemoglobin; Ht: haematocrit; Plt: platelet; MCV: mean corpuscular volume; MCH: mean corpuscular haemoglobin; MCHC: mean corpuscular haemoglobin concentration. Means ± SE. Kruskal-Wallis test: Significant differences among Control, MCI, and Dementia groups are indicated.

#### Blood enantiomeric proportions and their diagnostic values with regard to cognitive decline

Diagnostic values (sensitivity, specificity, area under the curve (AUC), 95% confidence interval (95% CI), and cut-off value) were determined by drawing receiver-operating characteristics (ROC) curves to verify the usefulness of blood enantiomeric proportion as a potential predictor for cognitive decline (Table 4). In the overall cohort, ROC analysis revealed that four proportions (D-Pro, D-Ser, D-Pro × D-Ser, and D-Pro × D-Ser/D-Ala) for all Control vs. MCI showed modest AUC (0.60–0.75), whereas four proportions (D-Ala, D-Pro × D-Ala, D-Ser × D-Ala, and D-Pro × D-Ser × D-Ala) exhibited low AUC (<0.60). The multiplied proportion of D-Pro × D-Ser had the highest AUC (0.70) with modest sensitivity (66%) and good specificity (74%) at the optimal cut-off value (0.30). In comparison, the ROC analysis revealed that five proportions (D-Pro, D-Ser, D-Pro × D-Ser, D-Pro × D-Ser × D-Ala, and D-Pro × D-Ser/D-Ala) for all Control vs. Dementia showed modest AUC, whereas three proportions (D-Ala, D-Pro × D-Ala, and D-Ser × D-Ala) exhibited low AUC. The proportion of D-Pro × D-Ser/D-Ala exhibited the highest AUC (0.73) with good sensitivity (83%) and modest specificity (70%) at the optimal cut-off value (0.85). Overall, these results indicated that blood enantiomeric proportion including D-Pro might therefore serve as a predictor for cognitive decline.

**Table 4:** Clinical values of blood enantiomeric proportions obtained in the Control vs. MCI or Dementia groups (all participants).

Group	Enantiomeric proportion	Sensitivity (%)	Specificity (%)	AUC	95% CI	*p*-value	Cut-off
**Control vs. MCI**	D-Pro, %D	57	78	0.66	0.593–0.717	<0.001	0.41
	D-Ser, %D	60	60	0.60	0.533–0.661	0.003	0.91
	D-Ala, %D	57	48	0.53	0.468–0.599	0.322	0.40
	D-Pro × D-Ser, %D^2^	66	74	0.70	0.645–0.763	<0.001	0.30
	D-Pro × D-Ala, %D^2^	50	58	0.55	0.480–0.611	0.170	0.17
	D-Ser × D-Ala, %D^2^	43	64	0.51	0.440–0.572	0.855	0.34
	D-Pro × D-Ser × D-Ala, %D^3^	50	62	0.57	0.500–0.631	0.047	0.18
	D-Pro × D-Ser / D-Ala, %D	62	65	0.68	0.617–0.738	<0.001	0.71
**Control vs. Dementia**	D-Pro, %D	67	77	0.67	0.397–0.951	0.218	0.45
	D-Ser, %D	83	61	0.67	0.389–0.951	0.235	0.94
	D-Ala, %D	50	57	0.54	0.344–0.743	0.671	0.41
	D-Pro × D-Ser, %D^2^	67	83	0.70	0.399–0.992	0.198	0.50
	D-Pro × D-Ala, %D^2^	83	54	0.57	0.362–0.778	0.500	0.16
	D-Ser × D-Ala, %D^2^	83	53	0.54	0.328–0.756	0.699	0.45
	D-Pro × D-Ser × D-Ala, %D^3^	67	65	0.62	0.390–0.859	0.299	0.20
	D-Pro × D-Ser / D-Ala, %D	83	70	0.73	0.495–0.960	0.055	0.85

All participants: Control (N = 150, MMSE: 30–27), MCI (N = 149, MMSE: 26–21), Dementia (N = 6, MMSE: 20–0). MCI: mild-cognitive-impairment.

For the matched cohort, healthy controls that exhibited an MMSE score of 30 were extracted to compare with the MCI and Dementia groups. Similarly, diagnostic values were determined by drawing ROC curves to verify the usefulness of blood enantiomeric proportion as a potential predictor for cognitive decline (Table 5). The ROC curves were drawn by plotting the proportion of sensitivity vs. the proportion of 1 − specificity (Supplementary Fig. 4). In the matched cohort, the ROC analysis revealed that the multiplied proportion of D-Pro × D-Ser for selected Control vs. MCI showed the highest AUC (0.80) with modest sensitivity (70%) and good specificity (84%) at the optimal cut-off value (0.30), whereas six proportions (D-Pro, D-Ser, D-Ala, D-Pro × D-Ala, D-Ser × D-Ala, and D-Pro × D-Ser × D-Ala) exhibited modest AUC and the proportion of D-Pro × D-Ser/D-Ala demonstrated low AUC. In comparison, the ROC analysis revealed that two proportions (D-Pro × D-Ala, D-Pro × D-Ser × D-Ala) for selected Control vs. Dementia showed good AUC ( > 0.75), whereas six proportions (D-Pro, D-Ser, D-Ala, D-Pro × D-Ser, D-Ser × D-Ala, and D-Pro × D-Ser/D-Ala) exhibited modest AUC. The multiplied proportion of D-Pro × D-Ser × D-Ala exhibited the highest AUC (0.80) with good sensitivity (83%) and good specificity (80%) at the optimal cut-off value (0.15). Therefore, the usefulness of blood enantiomeric proportion, including D-Pro as a predictor for cognitive decline, was also accepted in the matched cohort.

**Table 5:** Clinical values of blood enantiomeric proportions obtained in the Control vs. MCI or Dementia groups (selected participants).

Group	Enantiomeric proportion	Sensitivity (%)	Specificity (%)	AUC	95% CI	*p*-value	Cut-off
**Control vs. MCI**	D-Pro, %D	57	87	0.71	0.639–0.789	<0.001	0.41
	D-Ser, %D	58	57	0.60	0.519–0.681	0.016	0.91
	D-Ala, %D	68	55	0.67	0.589–0.743	<0.001	0.31
	D-Pro × D-Ser, %D^2^	70	84	0.80	0.723–0.877	<0.001	0.30
	D-Pro × D-Ala, %D^2^	74	58	0.72	0.642–0.792	<0.001	0.10
	D-Ser × D-Ala, %D^2^	68	58	0.67	0.598–0.752	<0.001	0.26
	D-Pro × D-Ser × D-Ala, %D^3^	65	68	0.73	0.653–0.802	<0.001	0.10
	D-Pro × D-Ser/D-Ala, %D	57	53	0.55	0.465–0.637	0.246	0.82
**Control vs. Dementia**	D-Pro, %D	67	87	0.72	0.457–0.987	0.100	0.45
	D-Ser, %D	83	58	0.67	0.381–0.955	0.251	0.94
	D-Ala, %D	67	68	0.68	0.429–0.924	0.163	0.41
	D-Pro × D-Ser, %D^2^	67	90	0.73	0.448–1.003	0.112	0.50
	D-Pro × D-Ala, %D^2^	83	80	0.77	0.543–0.996	0.020	0.16
	D-Ser × D-Ala, %D^2^	83	78	0.73	0.471–0.995	0.080	0.45
	D-Pro × D-Ser × D-Ala, %D^3^	83	80	0.80	0.553–1.042	0.026	0.15
	D-Pro × D-Ser/D-Ala, %D	67	68	0.62	0.361–0.878	0.365	1.19

Selected participants: Control (N = 60, MMSE: 30), MCI (N = 149, MMSE: 26–21), Dementia (N = 6, MMSE: 20–0).

## Discussion

In this study, a new identification method of chiral metabolomics for detecting a slight difference in the amount of chiral amino acids was developed to examine cognitive decline as a function of the detected difference. Additionally, a chiral tandem LC-MS/MS system was developed for the high-sensitivity, even at sub-femtomole levels, and high-selectivity determination of chiral amino acids, requiring approximately 20 min per sample. Compared with conventional methods^17–20^, this system was adopted to separate the molecular species of enantiomeric amino acids (all proteinogenic amino acids and non-proteinogenic amino acids such as Cit and Orn) with high-throughput for application to blood samples in addition to chiral amino acid standards. A previous study showed that chromatographic separation of DL-Ala, DL-Asp, and DL-Ser could be achieved by AQC derivatisation using a single ZWIX column^21^. However, the mechanism of such enantiomeric separation was not fully elucidated. Hydrogen bonding, electrostatic interactions, and π-π interactions have been described as interactions arising in an anion exchange type chiral column for enantiomer separation^22^. These three interactions may contribute to the analytical systems in the present study. When focusing on the separation of molecular species using a single QN-AX column, amino acids were eluted in the order of basicity, neutrality, and acidity. Therefore, ionic interactions between a free carboxyl group in an amino acid and the chiral stationary phase of the QN-AX column are considered to contribute to the separation of molecular species. In addition, hydrogen bonding and π-π interactions between AQC and the chiral stationary phase of the ZWIX (+) column may also contribute to enantiomer separation.

The enantiomeric proportions of D-Ser, D-Ala, and D-Pro in each amount of (D + L) detected in human blood were approximately 0.8–2.0%, 0.1–0.8%, and 0.1–0.8%^23–26^, respectively. These did not markedly differ from those determined in a previous study using two-dimensional liquid chromatography^18^ with healthy male plasma (1.65%, 0.54%, and 0.28%, respectively), while the enantiomeric proportion of D-Ser detected in this study was somewhat lower than that detected in previous reports, probably due to sex differences. Notably, the chiral tandem LC-MS/MS system developed in the present study was capable of separating the amino acids as well as their chiral species in a short time (within approximately 20 min) with high-precision and without solid-phase extraction upon application to blood samples.

We subsequently determined whether chiral amino acid levels might serve as a new blood biomarker for early diagnosis of cognitive decline. It was found that the proportion of D-Pro (D-Pro/ (D-Pro + L-Pro)) in the blood of the MCI group was higher than that in the Control group. In addition, when the proportion of D-Ser was combined with that of D-Pro (multiplied proportion: D-Pro × D-Ser), the correlation with early cognitive decline, as classified using the widely adopted cognitive function test MMSE, was improved. Moreover, the results of the multiplied proportion showed a high correlation with early cognitive decline in a cross-sectional study. No significant differences were observed by only L-amino acids, which are abundant in biological samples. Therefore, we assume that it is difficult for us to describe life science without mentioning trace D-amino acids.

Although D-Ser is suggested to become a promising diagnostic biomarker for AD because of the low amount of D-Ser in the brain and plasma of AD model mice in comparison with that of the healthy control model^27,28^, a clear correlation between D-Ser levels in human serum and cognitive function has not been reported^29^. Conversely, in the present study, we found that the amount of D-Ser gradually increased along with cognitive decline. This discrepancy may be a result of potential differences in the chiral metabolic pathway between mouse and human; thus, further assessment will be required to confirm that D-Ser in human blood is suitable as a potential biomarker for cognitive decline. However, D-Ser and D-Asp are the two most common isomerization residues found in AD tissue^30^. We also tried to find any correlations of D-Asp to cognitive decline in addition to D-Ser and D-Pro, but D-Asp was not detected from human blood as with previous reports^18^.

The present study demonstrated that the proportion of D-Pro was strongly associated with early cognitive decline. Consistent with this, serum amyloid P components have been shown to be depleted when a novel palindromic bis-D-proline drug was prescribed^31^. In addition, D-Pro is suggested to be related to brain-gut interactions through intestinal flora^32^ and to brain-mouth interactions through oral flora^33^. Based on these results, we suggest that D-Pro is likely to be involved with the selective clearance of amyloid beta in life systems. Moreover, we anticipate that the proportion of D-Pro may become a new brain health index in connection with cognitive function. Furthermore, with regard to chiral metabolism, D-Pro is decomposed and discharged by D-amino acid oxidase (DAO, EC 1.4.3.3)^34^. DAO catalyses the stereospecific oxidative deamination of the D-form of neutral amino acids, generating neurotoxic metabolites. Notably, the catalytic amount of DAO was reported to be correlated with the severity of cognitive decline^35^. Therefore, the relationship between D-Pro and DAO in cognitive decline should be clarified in further studies.

Several limitations of this study should be acknowledged. First, additional cohort studies should be conducted not only in cross-sectional but also in longitudinal format to confirm the timeline of cognitive decline. Second, to investigate sex differences, a study focused on a male population should be carried out. Third, as cognitive function was assessed only using MMSE in the present study, detailed categorisations on the basis of comprehensive cognitive assessments are needed to complement our findings. Fourth, the number of participants with dementia was limited. Further studies with larger numbers of patients with dementia are needed to strengthen our findings. Fifth, the influences of DAO and racemase should be examined. Nevertheless, our findings support that the blood enantiomeric proportion may represent a new brain health index in addition to L-amino acid profiles and provide useful information regarding cognitive decline. Lastly, more rapid analytical methods with better separation for chiral amino acids, particularly, D-Pro and D-Ser, should be developed to apply for more rapid diagnosis of cognitive decline.

In conclusion, we developed a new chiral tandem LC-MS/MS system for chiral amino acid metabolomics and performed correlation analysis between cognitive function and blood enantiomeric proportions using a cross-sectional cohort of elderly women. We found that the proportion, including D-Pro, was correlated with early cognitive decline. The accuracy of this biomarker was improved by the combination with D-Ser proportion. The identification method of chiral amino acids developed in this study might be applicable to practical medical evaluations for monitoring the early risk of cognitive decline with high accuracy and high throughput.

## Methods

### Analytical method development for chiral amino acids using chiral tandem LC-MS/MS

#### Reagents

Standards (chiral proteinogenic amino acids: DL-Ala, DL-Arg, DL-Asn, DL-Asp, DL-Cys, DL-Gln, DL-Glu, DL-His, DL-Ile, DL-Leu, DL-Lys, DL-Met, DL-Phe, DL-Pro, DL-Ser, DL-Thr, DL-Trp, DL-Tyr, DL-Val; chiral non-proteinogenic amino acids: DL-Cit, DL-*allo*-Ile, DL-Orn, DL-*allo*-Thr; achiral proteinogenic amino acid: Gly) were purchased from Tokyo Chemical Industry Co., Ltd. (Tokyo, Japan). An internal standard (DL-alanine-2,3,3,3-d4: DL-d4-Ala) was purchased from Santa Cruz Biotechnology (Dallas, TX, USA). For preparation of the mobile phase and sample pre-treatment, methanol plus (MeOH) for LC/MS and distilled water plus (H_2_O) for LC/MS were purchased from Kanto Chemical Co., Inc. (Tokyo, Japan). Formic acid (FA) for LC/MS was purchased from Fujifilm Wako Pure Chemical Corporation (Osaka, Japan). Ammonium formate (AF) for LC/MS was purchased from Thermo Fischer Scientific (Waltham, MA, USA). For pre-column derivatisation, the AccQ-Tag Ultra Derivatization Kit (a highly reactive amine derivatising reagent (AQC), borate buffer, and acetonitrile) was purchased from Waters Corporation (Milford, MA. USA).

#### Preparation of standard solutions and sample pretreatment

To prepare standard solutions, 10 μL of 10 nmol/L mixture solution (DL-Ala, DL-Arg, DL-Asn, DL-Asp, DL-Cit, DL-Cys, DL-Gln, DL-Glu, Gly, DL-His, DL-Ile, DL-*allo*-Ile, DL-Leu, DL-Lys, DL-Met, DL-Orn, DL-Phe, DL-Pro, DL-Ser, DL-Thr, DL-*allo*-Thr, DL-Trp, DL-Tyr, DL-Val, and DL-d4-Ala dissolved in 0.2 mol/L borate buffer (pH 8.9)) was mixed with 70 μL of 0.2 mol/L borate buffer (pH 8.9). Then, 20 μL of 10 mmol/L AQC solution (AQC reagent dissolved in acetonitrile) was added and mixed immediately. Subsequently, the mixture was heated at 55 °C for 10 min. For sample pre-treatment, 50 μL of human serum was mixed with 450 μL of 90% (v/v) methanol aqueous solution and centrifuged at 2130 × *g* for 10 min at 4 °C to remove proteins. Then, 10 μL of the supernatant was mixed with 70 μL of 0.2 mol/L borate buffer (pH 8.9), to which 20 μL of 10 mmol/L AQC solution was added and mixed immediately. The mixture was heated at 55 °C for 10 min. The reaction solution was then subjected to chiral tandem LC-MS/MS systems.

#### Chiral tandem LC-MS/MS analysis

Chiral tandem LC-MS/MS analysis was performed using the Exion LC System (AB Sciex, Redwood City, CA, USA) connected to a linear ion trap mass spectrometer (QTRAP6500^+^, AB Sciex) with ion source of electrospray ionization in MRM mode. Sciex Analyst (version 1.6.3) and MultiQuant (version 3.0.2) software were used for system control and data processing. The Exion LC System consisted of a binary pump, degasser, autosampler, and column oven. The interface parameters were optimized under the following conditions: curtain gas (CUR): 30 psi; ionspray voltage (IS): 4500 V; temperature (TEM): 600 °C; ion source gas1 (GS1): 80 psi; ion source gas2 (GS2): 80 psi; collision gas (CAD): 10. The ionspray position was set at default position (length: 3 mm, width: 7 mm). Protonated molecule ([M + H]^+^) and AQC-derived ion (*m/z* = 171) were selected as a precursor ion and a product ion, respectively. The MS parameters for detecting chiral amino acids were as described in the results section. Simultaneous separation of chiral amino acids was performed using Chiralpak QN-AX (first chiral column: 2.1 mm i.d. × 150 mm, 5 μm particles) (Daicel CPI, Osaka, Japan) and Chiralpak ZWIX (+) (second chiral column: 3.0 mm i.d. × 150 mm, 3 μm particles) (Daicel CPI) connected in series or Chiralpak QD-AX (first chiral column: 2.1 mm i.d. × 150 mm, 5 μm particles) (Daicel CPI) and Chiralpak ZWIX(−) (second chiral column: 3.0 mm i.d. × 150 mm, 3 μm particles) (Daicel CPI) connected in series as analytical columns. Column temperature was kept at 45 °C in the tandem chiral column method using QN-AX and ZWIX (+) columns. Alternatively, column temperature was kept at 40 °C in the tandem chiral column method using QD-AX and ZWIX (−). The mobile phase consisted of 90% (v/v) methanol aqueous solution containing 0.1% (v/v) FA and 55 mM AF. The injection volume was 5 μL, and the flow rate was 0.25 mL/min in isocratic conditions. The enentiomeric proportion was calculated as a percentage of D-form to total (D + L) concentration based on the peak area.

### Cognitive function and correlational analysis of blood chiral amino acid proportions in community-dwelling elderly individuals

#### Participants

The study population comprised 305 females (65–80 years old) who live in the Itabashi ward of Tokyo. Written informed consent was obtained from the subjects after being fully informed regarding the details and methods of this study. This research protocol was approved by the Clinical Research Ethics Committee of the Tokyo Metropolitan Institute of Gerontology (approval number: 929) and conformed with the Helsinki Declaration (ethics committee approval number: K38).

#### Blood sampling and analysis

The blood sample was collected from the forearm median vein and centrifuged at 2130 × *g* for 10 min at 4 °C to obtain serum based on a conventional method^36^. After centrifugation, the serum sample was stored at −80 °C until needed for measurements. The serum chiral amino acid proportion, creatinine; total cholesterol; HDL-cholesterol; triglyceride; albumin; white blood cell; red blood cell; haemoglobin; haematocrit; platelet; mean corpuscular volume; mean corpuscular haemoglobin; mean corpuscular haemoglobin concentration, and HbA1c were measured. Chiral tandem LC-MS/MS analysis was carried out in the above conditions and the enantiomeric proportion [D/(D + L) × 100] (%D) of each molecular species was obtained based on the peak area ratio.

#### Cognitive function assessment

MMSE was chosen as the cognitive function assessment for the subjects. MMSE consists of a series of questions that are divided into the eleven categories (date and time, place, memory, attention, recall, designation, repetition, understanding, reading, writing, and drawing). In this study, we classified the subjects into three groups based on MMSE score as follows: healthy control (30–27 points: Control group), suspected MCI (26–21 points: MCI group), and suspected dementia (20 points or less: Dementia group)^37^.

#### Statistical analyses

All clinical characteristics were presented as the means ± SE. All statistical analyses were performed using Excel statistical software (BellCurve for Excel, Social Survey Research Information Co., Ltd., Tokyo, Japan). All mean values between groups were compared using the Mann-Whitney U test for two groups or the Kruskal-Wallis test for three groups. ROC curves^38^ were used to verify the diagnostic value (cut-off value, AUC, sensitivity, specificity, and 95%CI) of enantiomeric proportions. A *p*-value less than 0.05 was considered statistically significant.

## Supplementary information

Supplementary Information.

## Data Availability

The datasets used and analysed during the current study are available from the corresponding author on reasonable request.
