# Supplementary material for: Development of a cognitive function marker based on D-amino acid proportions using new chiral tandem LC-MS/MS systems
Source: Sci Rep. 2020 Jan 21;10:804. doi: 10.1038/s41598-020-57878-y (PMC6972825; doi:10.1038/s41598-020-57878-y)
Supplement: Supplementary file 1 — Supplementary Information. [file 41598_2020_57878_MOESM1_ESM.pdf]

Development of a cognitive function marker based on D-amino acid proportions using new chiral tandem LC-MS/MS systems

Ren Kimura[1*], Hisashi Tsujimura[1], Masaru Tsuchiya[1], Satoko Soga[2], Noriyasu Ota[2], Atsushi Tanaka[3] & Hunkyung Kim[4]

Supplementary Information

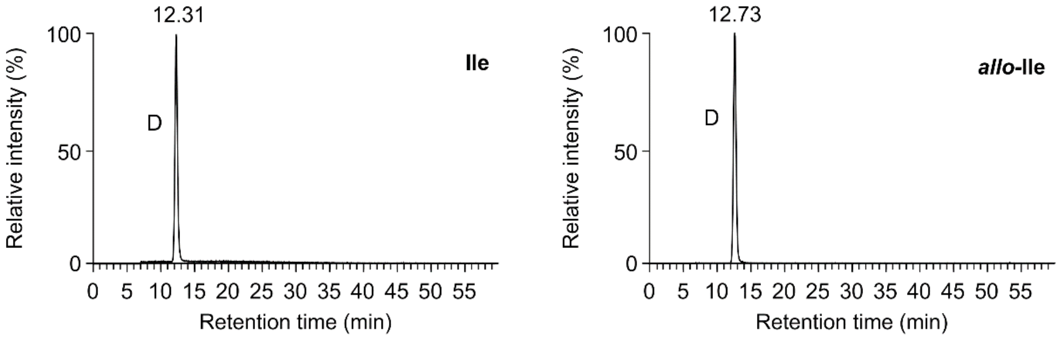

Supplementary Figure 1. Chromatograms of D-Ile and D-*allo*-Ile standards obtained using chiral tandem LC-MS/MS (QD-AX and ZWIX (−)).

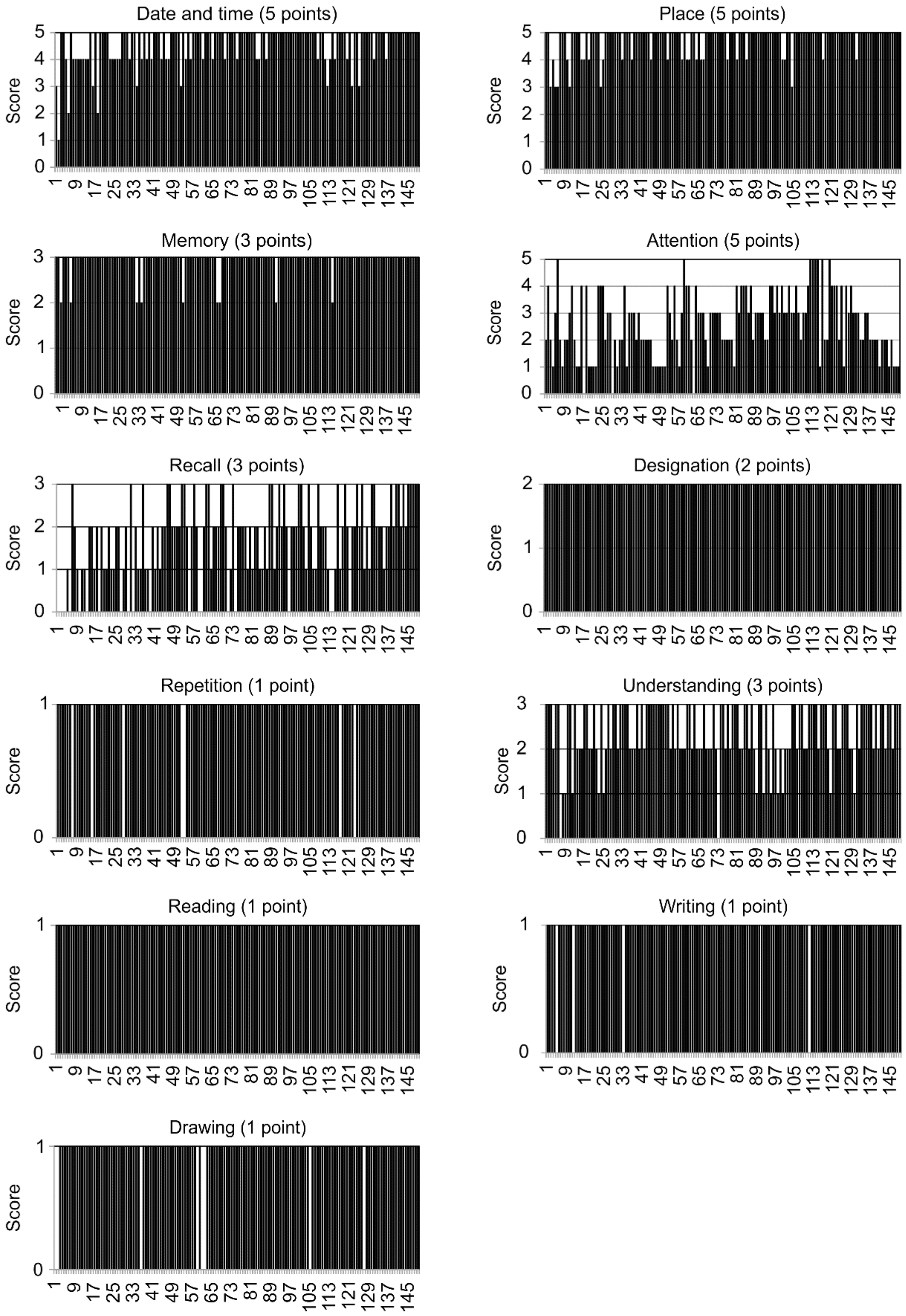

Supplementary Figure 2. Details of MMSE scores obtained in the MCI group (N = 149).

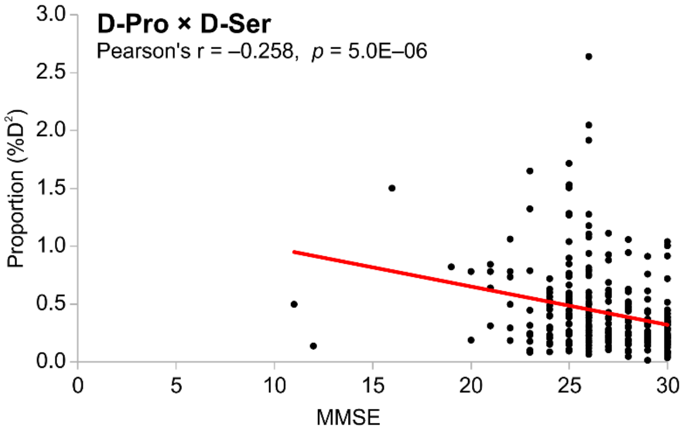

Supplementary Figure 3. Correlation between Mini-Mental State Examination (MMSE) score and multiplied proportion (D-Pro × D-Ser).

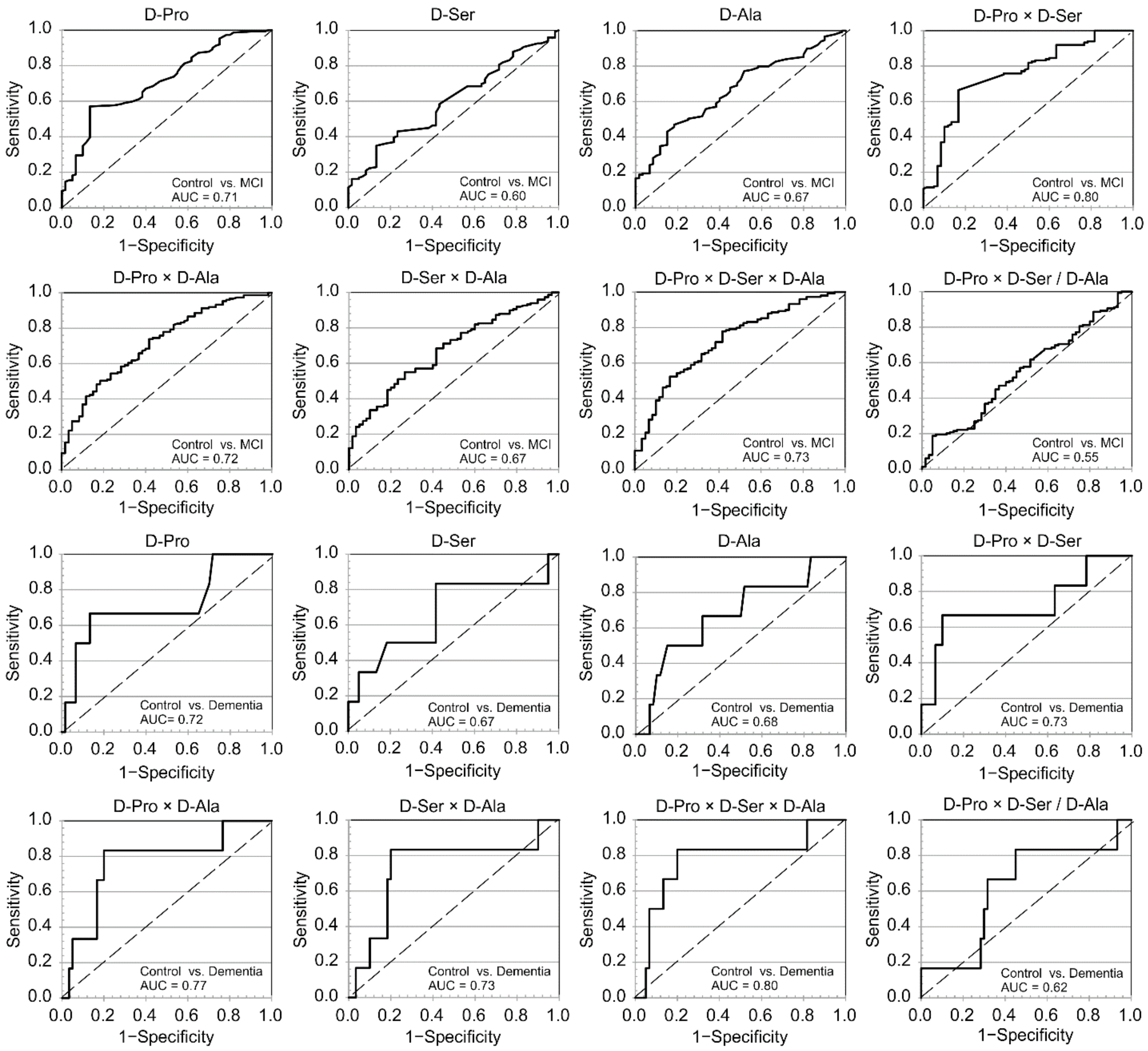

Supplementary Figure 4. ROC curve analysis of blood enantiomeric proportions obtained for the Control vs. MCI or Dementia groups. AUC, area under the curve.
